# Failure of Primary Distal Biceps Repair After Cortical Button Fixation With Whipstitch Technique: A Root Cause Analysis

**DOI:** 10.7759/cureus.21254

**Published:** 2022-01-14

**Authors:** Naga Cheppalli, Sreenivasulu Metikala, Eric Leung, Dustin L Richter

**Affiliations:** 1 Orthopaedic Surgery, University of New Mexico School of Medicine, Albuquerque, USA; 2 Orthopaedics, Virginia Commonwealth University School of Medicine, Richmond, USA; 3 Orthopaedics, University of New Mexico School of Medicine, Albuquerque, USA

**Keywords:** suture stiffness, distal biceps rupture, cheese-wiring, whipstitch, cortical button technique, revision operation, distal biceps tendon repair

## Abstract

Rerupture after cortical button fixation and whipstitch suture technique is a rare complication of distal biceps tendon repair. The tendon-bone fixation construct can fail for various reasons, including cortical breach, pull out or disengagement of a cortical button, suture breakage, or knot slippage. Occasionally, a cut-through of the tendon substance by the high-tensile strength suture material, called the ‘cheese-wire’ effect, can happen, especially with premature loading during the early postoperative period. The clinical presentation is more subtle, and the rerupture may go unnoticed. A high index of suspicion and a low threshold for ordering a magnetic resonance imaging (MRI) scan are necessary for a prompt diagnosis and early treatment. We describe the management of a reruptured distal biceps in an active male that happened in the early postoperative period, along with a critical analysis of the failure pattern and potential preventive measures.

## Introduction

Multiple fixation methods have been described to repair a distal biceps tendon rupture, including bone tunnels, suture anchors, interference screws, cortical buttons, or a combination of the above devices. The cortical button (CB)-based technique offers a stronger fixation with a significantly greater load to failure than the other techniques [1]. Tendon rerupture is a major postoperative complication with an incidence of 1.6-4% [2]. Most of the ruptures occur within the first three weeks of surgery because of limited patient compliance, which may cause excessive forces across the repaired construct [3]. Although a rerupture after primary repair with the cortical button is rare, the causes and modes of failure have not been fully explored in the literature. We present a case report analyzing the grounds of a reruptured distal biceps tendon after primary repair using a CB with whipstitch suture fixation technique and its surgical management with one-year follow-up results.

## Case presentation

A 60-year-old male, a retired veteran, presented to our clinic with an isolated full-thickness distal biceps tendon tear in the non-dominant left upper extremity after attempting to grab a falling motorbike. He had no prior history of elbow pain or injury. He was a non-smoker and was not on any steroid medication. On clinical examination, the patient demonstrated a ‘reverse Popeye’ sign and positive hook test. The MRI and ultrasound examination revealed a full-thickness distal biceps avulsion with retraction until the junction of the middle and distal thirds of the humerus. After a thorough evaluation, we recommended surgery, discussing the risk-benefit profile, expectations, and rehabilitation plan. He underwent surgery five days post-injury through a standard anterior approach [4].

Description of the primary surgical repair technique

A 5-cm longitudinal incision was made along the medial border of the mobile wad of Henry starting just distal to the elbow crease. The lateral antebrachial cutaneous nerve was identified in the subcutaneous plane, isolated, and protected throughout the surgery. An additional 1-cm transverse incision was created in the lower arm at the retracted biceps tendon stump level, which was localized by preoperative ultrasound. The distal stump was identified, the frayed ends were debrided and sized to 7 mm. A #2 FiberLoop® (Arthrex, Inc., Naples, Florida) was whipstitched through the distal 1-inch of the tendon stump (SpeedWhip™ Technique, Arthrex, Inc., Naples, Florida), taking care to lock the suture-fixation construct by reversing the final throw [5]. The tendon was then retrieved into the principal incision through a subcutaneous tunnel across the elbow. The two strands of the FiberLoop suture were threaded through a CB and kept ready for repair. Attention was now turned to the radial tuberosity footprint that was exposed by careful dissection. A 3-mm bicortical tunnel was made into the ulnar aspect of the radial tuberosity, keeping the forearm in maximum supination. A 7-mm socket was made in the near cortex using a cannulated reamer followed by thorough irrigation of bone debris. The CB was then inserted into the drill hole and flipped on the far cortex using the inserter. Sequentially, both suture strands were tensioned to dock the tendon stump into the prepared bone socket. The suture stands were finally passed through the tendon stump at the near cortex with a free needle and tied with a surgical knot. No gap at the repair site was noted with the elbow motion and forearm rotations. The wound was closed in layers, dressed, and the arm was kept in a sling. The patient was discharged home on the same day with non-weight-bearing precautions. Instructions were given on early progressive passive Range of Motion (ROM) exercises of the elbow and forearm for up to six weeks, followed by active ROM exercises. The graduated resistance training was planned to begin after three months.

**Figure 1:**
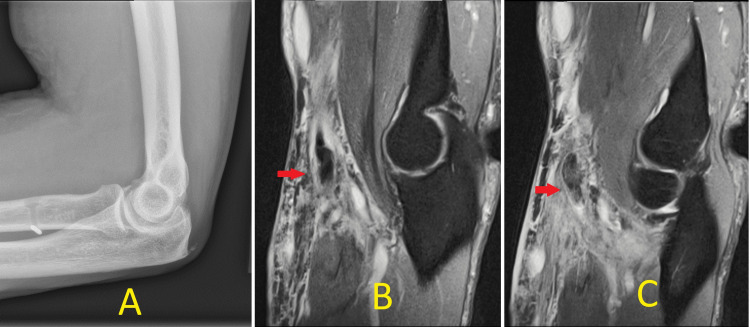
Imaging studies of the left elbow. The lateral radiograph (A) shows post-surgical changes with intact cortical button. MRI coronal T2-weighted slices (B, C) showing proximal retraction (arrow) of repaired distal biceps stump.

Unfortunately, on the seventh postoperative day, he developed sudden onset of severe pain at the surgical site after an inadvertent attempt to pull a sheet that was stuck. Clinical examination revealed fullness and tenderness in the antecubital fossa. The elbow motion, which was otherwise progressing well until the above incident, was limited due to pain. The surgical incision was unremarkable with intact sutures. It was also possible to hook the examiner's index finger around the lateral edge of the biceps tendon. The plain radiographs of the elbow revealed an intact CB on the far cortex of the radius with no signs of an acute fracture or cortical breach. Ultrasound examination was not possible due to significant tenderness and swelling. An urgent magnetic resonance imaging (MRI) scan of the elbow without contrast was ordered, which demonstrated loss of continuity with a 3-cm proximal retraction of the distal biceps tendon (Figure 1).

After discussing the risks and benefits of the management options and rehab protocol, the patient elected to undergo revision surgery.

Description of the revision surgery

**Figure 2:**
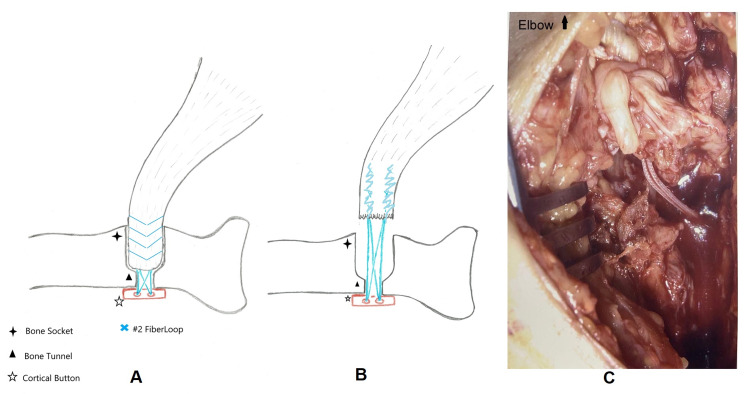
'Cheese-wire' effect of FiberLoop® suture strands. Illustration (A) of cortical button and intact whipstitch fixation construct during primary surgery. Illustration (B) and intraoperative image (C) of cheese-wiring behavior of FiberLoop with no suture breakage.

After standard surgical preparation, the old surgical incision was re-opened. The lateral antebrachial cutaneous nerve, once again, was identified and protected throughout. The discontinuity of the previous repair was noted with the tendon stump pulled away from the radial tuberosity footprint. The CB was intact at the far cortex with no migration or cortical breach. Although the FiberLoop suture strands were in continuity, they were found to cut through the tendon tissue resulting in a cheese-wire effect (Figure 2).

After removing all the previous suture material and CB, attention was turned to a revision repair using a combination of a new CB and suture anchor. First, the distal 1-inch tendon stump was fixated by a 1.3 mm SutureTape (Arthrex, Inc., Naples, Florida) in a locking Krackow stitch configuration [6]. Care was taken to preload each suture-pass along with cyclic loading of the final tendon-suture construct before threading the tape limbs through CB. The previous bone tunnel and socket were freshened and lavaged with copious saline. The tendon stump was now docked into the socket in the standard fashion, flipping the CB on the far cortex and sequential tensioning of both tape limbs. The fixation was then augmented by inserting a 1.9-mm intramedullary suture anchor (FiberTak®, Arthrex, Inc., Naples, Florida) 1 cm proximal to the CB fixation, and its two tape limbs were passed through the tendon tissue in a horizontal mattress fashion. The elbow ROM and forearm rotations confirmed the stability of the final repair with firm contact at the tendon-bone interface. The wound was then irrigated with copious normal saline removing all the bone debris. Layered wound closure was performed in a standard fashion. The extremity was placed in a long-arm posterior splint, and the patient was discharged home the same day. The splint and sutures were removed at two weeks, and the arm was placed in a sling.

**Figure 3:**
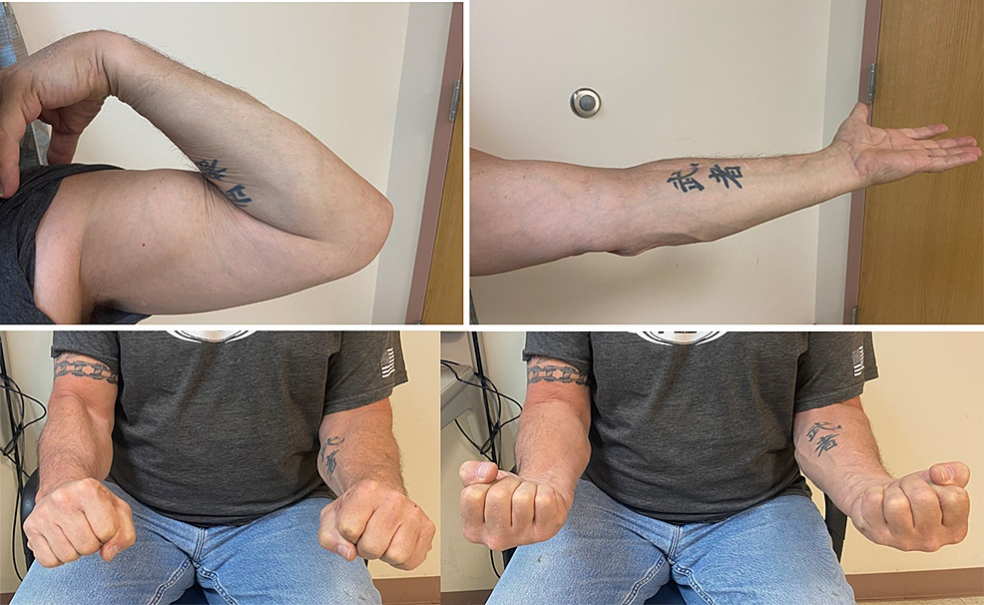
Postoperative clinical images at one-year follow-up. Left elbow (A) showing 5-130 degrees of ROM and (B) symmetrical forearm rotations.

Outpatient therapy was initiated, allowing passive ROM exercises as tolerated. At six weeks, the sling was discontinued to begin active ROM and isometrics. The resistance training started at three months and advanced in a gradual fashion. The patient resumed full duty with no restrictions at six months. The patient experienced numbness in the distribution of the superficial radial sensory nerve, which resolved after nine months. At the final one-year follow-up, the patient had no pain at the repair site and regained 5-130 degrees of elbow ROM, along with 90-degree supination and 70-degree pronation, same on both sides (Figure 3).

He continued to perform his duties with no restrictions, although a minimal reduction (grade 4+ power) in composite flexion-supination biceps strength was noted compared to the uninjured side. As per the variant of the Mayo Elbow Performance Index (MEPI), he achieved an excellent result with a final score of 94/100. The shortened version of the Disability of Arm, Shoulder and Hand Questionnaire (QuickDASH) score was 4.5/100 [7].

## Discussion

The diagnosis and prompt treatment of a distal biceps tendon rerupture, especially in the early postoperative period, can be challenging. This case study presented the management of a failed distal biceps repair after CB fixation with the whipstitch technique. In addition, a root cause analysis is presented, discussing the failure characteristics and preventive measures.

**Figure 4:**
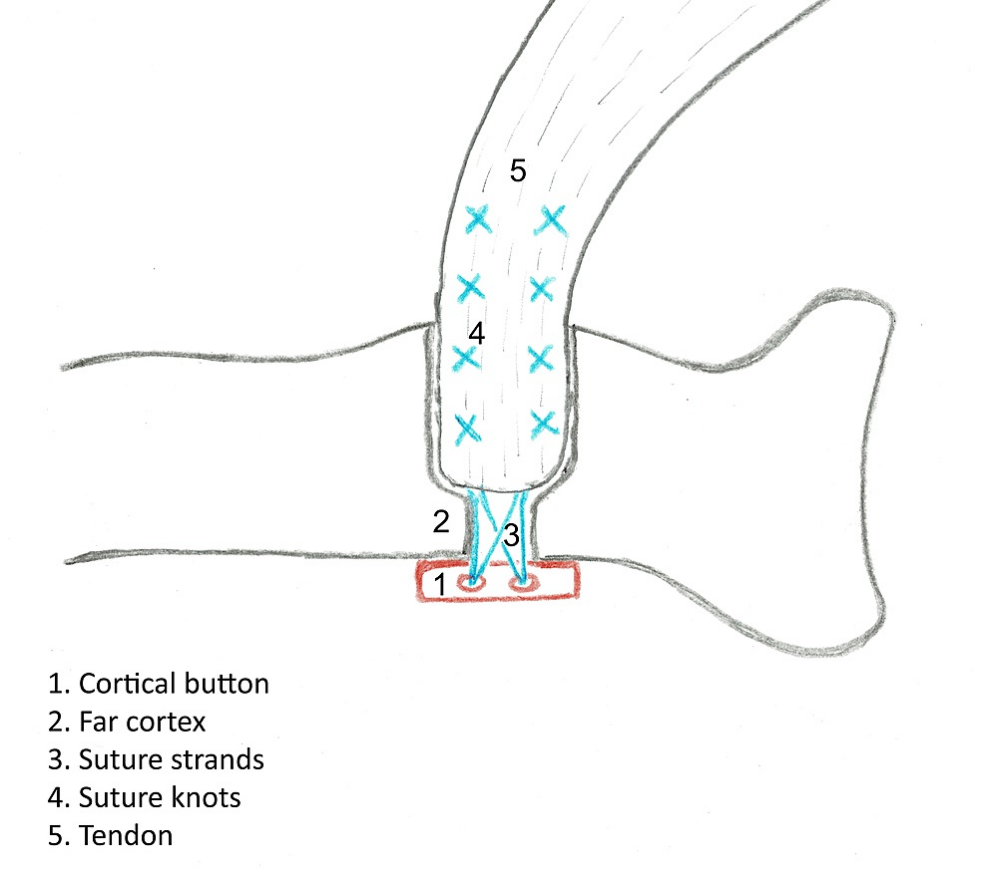
Possible sites of failure of distal biceps repair after cortical button fixation and high-tensile strength suture material.

There are multiple places where a CB fixation construct can mechanically fail. The sites, from distal to proximal, include the CB, far cortex, suture strands, suture knots, and tendon (Figure 4).

The common complications of CB fixation of distal biceps tendon repair include a breach at the far cortex, pull out of CB, or disengagement [8,9]. Strict adherence to the surgical technique and liberal use of intraoperative imaging can minimize these complications. Suture abrasion against the bone tunnel edges may lead to mechanical failure, especially when the tendon is not fully dunked to the far cortex [9,10]. Again, diligent practice of the standard tension slide technique with sequential tensioning of each suture limb ensures complete seating of the tendon stump and reduces the likelihood of suture damage. Also, preloading of each suture passes through the tendon substance, and cyclic loading of the final suture fixation construct can help address the suture creep and enhance overall stability. Suture knots can be another weak link in the fixation construct that can easily be overcome by learning a proper knot-tying technique. As a standard, at least five throws are necessary for a secure square knot, and when using a sliding knot technique, a minimum of three final reverse half hitches with alternating posts are recommended for knot security [11,12].

In our situation, there was a cut-through of the tendon substance by the high-tensile strength nonabsorbable (FiberLoop) suture stands with no failure detected elsewhere in the overall fixation construct. Such damage at the suture-tendon interface caused by the suture material without any suture breakage is called the 'cheese-wire' effect, often seen with high-strength suture materials. Notably, FiberLoop, a continuous loop of FiberWire® suture (Arthrex, Inc., Naples, Florida) made of a multi-strand long-chain ultra-high molecular weight polyethylene (UHMWPE), has the highest stiffness compared to other synthetic suture materials. The high-tensile strength of FiberWire, although minimizes suture breakage, is responsible for its tendon cheese-wiring behavior during cyclic loading or sudden eccentric loading, resulting in suture fixation failure [13,14].

Besides suture material, the suturing technique is critical to diminish the likelihood of failure at the suture-tendon interface. During the primary repair, we secured the distal end of the tendon substance by a whipstitch or the SpeedWhip Technique using a nonlocking premanufactured # 2 FiberLoop. It is a popular technique described for time efficiency and achieves uniform compression of the tendon tissue when applied as per the manufacturer's guidelines [15]. Despite these advantages, the whipstitch technique demonstrated higher elongation of the suture-tendon construct under preloading and cyclic loading phases compared to other suture configurations [16,17]. We speculate in our situation that the eccentric loading during the early postoperative period led to similar elongation of the tendon-suture interface and the stiffness of FiberWire caused the cheese-wiring of the tendon, ultimately resulting in functional construct failure.

During the revision surgery, we introduced Krackow locking stitch configuration using a SutureTape, an upgraded version of FiberWire suture with a flat braided construct having a greater mean load to failure [18]. Additionally, we inserted a suture anchor proximal to the CB construct, which helped to augment the repair, offload the tension on the CB's suspensory fixation, and also expand the footprint of the tendon-bone interface. Being an intramedullary design with a 1.9 mm unicortical hole, the above suture anchor fixation is less likely to have an intra- or postoperative fracture risk. We believe that the changes in suture material design and suturing technique along with double fixation (CB and suture anchor) allowed an overall satisfactory functional outcome after the revision surgery. Table 1 describes common suture-related failures and tips to minimize the complications.

**Table 1:** Summary of common suture-based failure patterns and potential solutions.

	Failure pattern	Solution
1	Suture breakage	High-strength synthetic material
2	Knot slippage (loop failure)	Hold the first knot with a hemostat or two-throw surgeon's knot
3	Knot failure	Square each knot with a minimum of five throws
4	Suture creep	Preload after every pass
5	Cheese-wiring	Intermediate stiffness locking suture with a 'tape' design

Finally, identifying a retear after distal biceps repair poses a diagnostic challenge. The symptoms can be subtle, and plain radiographs or ultrasound imaging offer little value. The treating physician should have a high index of suspicion based on the history and low threshold to order an MRI scan for a prompt diagnosis. Also, patient education on every detail of postoperative rehabilitation protocol is essential to avoid excessive loading of the repair construct during the initial healing phase. Immobilization in a temporary splint or hinged brace may be necessary for select patients with questionable compliance.

## Conclusions

The distal biceps repair using CB with whipstitch design may fail at the suture-tendon interface due to high-tensile strength suture material and nonlocking suture fixation technique. A thorough understanding of suture material characteristics and careful attention to each detail of the soft tissue fixation technique are critical aspects of repair to lessen the chances of mechanical failure. Patient education is also an integral component to prevent excessive loading of repair construct during the healing phase. Efficient detection of a retear is a diagnostic challenge that necessitates a high index of suspicion.
